# A Novel Real Time PCR Method for the Detection and Quantification of *Didymella pinodella* in Symptomatic and Asymptomatic Plant Hosts

**DOI:** 10.3390/jof8010041

**Published:** 2021-12-31

**Authors:** Adnan Šišić, Thomas Oberhänsli, Jelena Baćanović-Šišić, Pierre Hohmann, Maria Renate Finckh

**Affiliations:** 1 Department of Ecological Plant Protection, University of Kassel, 37213 Witzenhausen, Germany; mfinckh@uni-kassel.de; 2 Department of Crop Sciences, Research Institute of Organic Agriculture (FiBL), 5070 Frick, Switzerland; 3 Section of Organic Plant Breeding and Agrobiodiversity, University of Kassel, 37213 Witzenhausen, Germany; bacanovic@uni-kassel.de; 4 Sustainable Plant Protection Programme, Institute of Agrifood Research and Technology (IRTA), Av. Alcalde Rovira Roure, 177, 25199 Lleida, Spain; pierre.hohmann@irta.cat

**Keywords:** pea, foot and root rot, *Didymella pinodella*, *Phoma medicaginis* var. *pinodella*, qPCR, wheat, asymptomatic infections

## Abstract

*Didymella pinodella* is the major pathogen of the pea root rot complex in Europe. This wide host range pathogen often asymptomatically colonizes its hosts, making the control strategies challenging. We developed a real-time PCR assay for the detection and quantification of *D. pinodella* based on the *TEF-1 alpha* gene sequence alignments. The assay was tested for specificity on a 54-isolate panel representing 35 fungal species and further validated in symptomatic and asymptomatic pea and wheat roots from greenhouse tests. The assay was highly consistent across separate qPCR reactions and had a quantification/detection limit of 3.1 pg of target DNA per reaction in plant tissue. Cross-reactions were observed with DNA extracts of five *Didymella* species. The risk of cross contamination, however, is low as the non-targets have not been associated with pea previously and they were amplified with at least 1000-fold lower sensitivity. Greenhouse inoculation tests revealed a high correlation between the pathogen DNA quantities in pea roots and pea root rot severity and biomass reduction. The assay also detected *D. pinodella* in asymptomatic wheat roots, which, despite the absence of visible root rot symptoms, caused wheat biomass reduction. This study provides new insights into the complex life style of *D. pinodella* and can assist in better understanding the pathogen survival and spread in the environment.

## 1. Introduction

Root rot of pea is one of the main factors contributing to the decline of cultivated area of this important crop worldwide. The disease is caused by a complex of fungal pathogens with multiple pathogenic species from the genus *Fusarium* implicated as the common causal agents [1,2,3,4,5]. Besides *Fusarium* spp., *Aphanomyces euteiches* has been recognized as a devastating pathogen in North America and France, particularly when pea is grown in short rotations and wet soils [3,6]. In addition, species such as *Didymella pinodella* (syn. *Phoma pinodella*; *Phoma medicaginis* var. *pinodella*), *D. pinodes* (syn. *Mycosphaerella pinodes*), *Rhizoctona solani*, *Pythium* spp., and *Sclerotinia sclerotiorum* are also an important part of the disease complex [2,7,8,9,10]. Their prevalence and dominance may vary greatly depending on the geographical region and pedo-climatic conditions. 

In order to better understand their virulence and lifecycle, the detection and quantification of root pathogens are crucial [11,12]. However, pathogen identification often relies on laborious culture based morphological laboratory techniques and microscopy. Besides being time consuming, a high level of expertise is needed for accurate species level identification. As morphological features can vary depending on the environmental and cultural conditions, identification always remains somewhat subjective. Furthermore, often more than one single species of the complex colonize the roots simultaneously and there is growing evidence that the culture media tend to select for fast growing and more competitive species seriously limiting this technique to evaluate species interactions *in planta*. The study of Zitnick-Anderson et al. [12], for example, showed that the detection of different *Fusarium* spp. from inoculated pea roots was always higher using molecular methods compared to traditional plating assays. They found that some species such as *F. avenaceum* and *F. acuminatum* were always over-represented via plating, while the majority of other species used in their study were under-represented or below detection level with cultural techniques alone. Similarly, Armstrong-Cho et al. [13] and Chatterton et al. [3] reported difficulties and often failure of cultural methods to detect *A. euteiches* in pea roots even when semi-selective media were used.

Similar challenges exist with isolation and identification of plant associated *Didymella* spp. For example, in Europe, *D. pinodella* is one of the most dominant pathogens associated with root rots of pea and faba bean [2,8,14]. Our recent study showed particular dominance of this species in roots of winter pea varieties in Germany, which were found surprisingly less frequently colonized by *Fusarium* spp. [14]. We also found *D. pinodella* in asymptomatic vetch, clover and wheat roots [5,15]. Overall, this wide host range pathogen is reported to infect at least 18 species in 14 plant genera [16]. Similar to *A. euteiches*, *Didymella* spp. are relatively slow growing fungi that are commonly overgrown by fast growers, requiring frequent sub-culturing. Furthermore, in addition to *D. pinodella*, the less frequent, closely related *D. pinodes* [2] and *D. lethalis* [17] were also reported as a part of the pea root rot complex, and are likely to co-occur on other grain legumes as well. The three *Didymella* species share many important cultural and morphological characteristics and discrimination of these pathogens based on the morphology alone can be difficult [18,19]. Moreover, the sexual morph of *D. pinodella,* which is at the moment only reported on agar media cultures, is morphologically very similar to *D. pinodes* and, if present in natural infections, is likely to be misidentified as the latter species [20]. In addition, due to probable recent evolutionary divergence from a common ancestor, *D. pinodella*, *D. pinodes* including the species *D. lethalis* are genetically very similar and exhibit a high level of inter species haplotype sharing [18,19]. Phylogenetic studies currently used for species level identification in *Didymellaceae* are inferred primarily from partial sequences of the internal transcribed spacer (*ITS*), the 28S rRNA (*LSU*), beta tubulin (*tub2*), RNA polymerase II the second largest subunit (*rpb2*) and the actin (*act*) gene regions [18,19,21]. None of these regions alone, however, is sufficiently informative to discriminate the three species. Thus, to achieve species level taxonomic resolution, comparisons of concatenated data sets of two or more different loci are needed. 

Previous attempts to develop a qPCR assay that can distinguish *D. pinodes* and *D. pinodella* in soil and plant samples were unsuccessful [22]. The authors targeted the *ITS* region and found that these two pathogens, including the species *D. lethalis* (observed in this study), were identical in this region. The goal of the present study was to develop a rapid and sensitive technique for the detection of *D. pinodella* and to validate it in two different host plant models. We further discuss possible implications of the results generated from the greenhouse inoculation experiments and the potential applicability of the developed qPCR assay in a wider ecological context.

## 2. Materials and Methods

### 2.1. Fungal Isolates

A total of 64 isolates representing 35 species were included in this study (Table 1). The isolates comprised 20 *Didymella*, 12 *Fusarium* and one species of each, *Boeremia*, *Juxtiphoma* (syn. *Phoma*) and *Paraphaeosphaeria*. Fifteen different *Didymella* spp., selected as phylogenetically close to *D. pinodella* based on the phylogenetic results of Chen et al. [18], were obtained from the Westerdijk Fungal Biodiversity Institute (KNAW, Utrecht, the Netherlands) culture collection. Isolates of the remaining species included in this study were obtained from the University of Kassel culture collection maintained at the Ecological Plant Protection Department. The isolates were originally recovered from symptomatic or asymptomatic roots of various field grown legumes with notable exceptions of one isolate of *D. macrostoma* and one of *Boeremia exigua* which, in addition to legume hosts, were recovered from a diseased apricot branch (*Prunus* sp.) (Table 1). All isolates in the internal University of Kassel culture collection were characterized morphologically, and their taxonomic identity was confirmed by sequencing the ITS, beta tubulin, actin and/or RPB2 genes for *Didymella* isolates and related species, and the *TEF-1 alpha* gene region for *Fusarium* spp. [5,15,17,23,24]. 

### 2.2. Gene Sequence Collection and Primer/Probe Set Design

Partial gene sequences of the β tubulin (*tub2*), actin (*act*), the RNA polymerase II the second largest subunit (*rpb2*), and the translation elongation factor 1 (*TEF-1 alpha*) were evaluated in silico for the presence of suitable regions to design primers and probes specific for *D. pinodella*. The *tub2* and *rpb2* gene sequences were retrieved from the publically accessible database of the TreeBASE previously deposited by Chen et al. [18] (accession number S20724). The *act* gene sequences were collected manually using the *tub2/rpb2* gene sequence strain numbers (see Table 1 in [18]) as search entries in the public database of the National Center for Biotechnology Information (NCBI). These three loci are commonly used to infer the relationships among the species in *Didymella* and related genera such as *Phoma*, *Boeremia*, *Aschochyta*, *Heterophoma*, and others [18]. Depending on the locus, the data set comprised 181–288 gene sequences that represented up to 188 different species. The *TEF-1 alpha* data set comprised 16 gene sequences only, representing three *Didymella* (seven sequences (n = 7) of the targeted *D. pinodella*, *D. pinodes* (n = 3) and *D. glomerata* (n = 1) and four *Ascochyta* species (i.e., one sequence of each *A. pisi*, *A. rabiei*, *A. fabae* f. sp. *viciae* and *Ascochyta* sp.) (Figure S1). There were no *TEF-1 alpha* sequences available for *D. lethalis*. The limited data availability in the NCBI database is because the *TEF-1 alpha* is not routinely used in the genetic analyses of the species in *Didymellaceae*.

Sequence alignments were constructed for each locus separately via Multiple Alignment using Fast Fourier Transform (MAFFT) [25]. Depending on the locus, our own generated sequence data were included in the alignments (Table 1). The Beacon Designer software (v.7.2.) was then used to identify candidate primers and corresponding probe sets specific for *D. pinodella*. Specific criteria for primer and probe selection were: (i) an annealing temperature range between 58 and 62 °C for the primers and 5–10 °C higher for the fluorogenic hydrolysis probe, (ii) length of 18–25 bases, (iii) G/C between 30% and 60%, (iv) an amplicon length of 70–200 base pairs, (v) the avoidance of hairpin and self-dimer formation and, (vi) a maximum distance between primer and probe of 20 bases. A total of seven sets of candidate primer pairs were designed in silico (Table S1). Of these, five were designed based on the beta tubulin sequence alignments, and two primer pairs from alignments of the *TEF-1 alpha* gene region. Due to the high level of sequence homology between the target *D. pinodella* and *D. pinodes* and *D. lethalis*, the *actin* and the *RPB2* gene sequences contained no informative regions to design candidate primers and/or probe sets.

### 2.3. Primer Specificity and qPCR Conditions

In the initial step, the target-specific primer screening of the seven primer pairs designed was performed without labeled probes using a SYBR Green based qPCR method on a 3 isolate/3 species exclusion panel. This panel was designed to include DNA extracts from three closely related species characterized by very low level of nucleotide polymorphisms in each of the target loci. Namely, the exclusion panel comprised species *D. pinodella* (FOEP 51.11581), *D. pinodes* (FOEP 51.11585), and *D. lethalis* (FOEP 51.11668) (Table 1). Each 15 µL reaction contained 7.5 µL SsoAdvanced™ Universal SYBR^®^ Green Supermix (Bio-Rad, Laboratories, Hercules, CA, USA), 1.5 µL of forward and reverse primer in two different final concentrations of 0.2 µM and 0.5 µM each, 1 µL of template DNA in two different final concentrations of 5 and 50 ng per reaction, and 5 µL of nuclease free water. PCR cycling conditions were as follows: initial denaturation and hot-start enzyme activation for 3 min at 98 °C, followed by 44 cycles of denaturation at 95 °C for 10 s, annealing in a temperature gradient set at 55–63 °C for 30 s and extension at 72 °C for 20 s. Following the melting curve and the quantification cycle data analysis, none of the primer pairs alone could discriminate the target from the non-target *Didymella* species.

In the second step, the mismatches in the sequence alignments of the probe-binding sites were evaluated for insertion of internal probes and a further increase of the assay specificity. Internal probe sequences of the targeted *D. pinodella* and the two non-target species *D. pinodes* and *D. lethalis* were identical in the five of the seven primers tested. Of these, four primer pairs targeted the *tub2* gene region and one primer pair targeted the *TEF-1 alpha* gene region. Of the remaining two primer pairs, *DpinodellaTub* and *DpinodellaTef*, the latter was selected as more promising candidate based on the higher presence of species specific single nucleotide polymorphisms and thus used for the insertion of a probe containing locked nucleic acid (LNA^®^) modified bases at the mismatch positions [26] (Table 2 and Table S1; Figure S1). This primer/probe set was again tested on the 3 isolate/3 species exclusion panel and its specificity further validated on a 54 isolate—35 fungal species panel in a qPCR assay. The validation panel included DNA extracts from a range of target, genetically related and some of the commonly occurring soil- and legume associated fungal species (Table 1). The selected primer pair and probe set underwent a final validation using DNA extracted from infected plant tissue generated in a greenhouse (see below).

### 2.4. Assay Conditions

The hybridization probe based qPCR mixture contained the following components: 7.5 µL of 2× SsoAdvanced Universal Probes Supermix (Bio-Rad), 1.5 µL forward and reverse primer (0.3 µM), 1 µL corresponding hydrolysis probe (0.1 μM), 1.5 µL of DNA template and nuclease free water to make the total volume of 15 µL. The template DNAs were used in the following concentrations: 5 and 50 ng µL^−1^ in the initial 3 isolate/3 species exclusion panel; stock DNAs (concentrations ranging from 11.3 to 1997 ng DNA mL^−1^), 10× diluted stock DNA or 100 ng μL^−1^ DNA in a 54 isolate/35 species validation panel and; 100 ng μL^−1^ standardized DNA extracted from greenhouse inoculated root tissue. As negative control samples served nuclease free water substituted for a DNA template or *D. pinodella*-free plant DNA sample obtained from non-inoculated treatments of greenhouse experiments. 

To optimize the qPCR conditions, the optimal primer annealing temperatures were determined with the initial 3 isolate/3 species exclusion panel in a temperature gradient from 54 to 62 °C achieving the highest amplification efficiency and the lowest quantification cycle (Cq) numbers needed for the discrimination of two serial dilutions, and finally, for the absence of cross-reaction with *D. pinodes* and *D. lethalis*. All reactions were run for 40–45 cycles; however, random presence of unspecific (off-target) signals were observed at Cq values above 35 which was set as a cut-off value. Moreover, the standards with the lowest DNA concentrations were able to reach the plateau during the first 35 cycles. Thus, for the assay reported here, the optimum qPCR conditions for amplifications were initial denaturation for 3 min at 98 °C, followed by 34 cycles of denaturation at 95 °C for 10 s and annealing, extension and measuring of fluorescent emission at 61 °C for 30 s. All PCR reactions (SYBR Green and hybridization probe based assays) were carried out in a BioRad CFX96 real-time PCR detection system (Bio-Rad Laboratories, Hercules, CA, USA). A minimum of two simultaneous or separate replicate reactions were performed for each sample to confirm the reproducibility of the results.

### 2.5. Production of Infected Plant Material

Final primer and probe set specificity tests for qPCR assay validation were performed by inoculating field pea cv. Santana and winter wheat cv. Achat with ten different *D. pinodella* isolates under greenhouse conditions. All ten isolates were tested on pea and eight were tested on wheat. The isolates were recovered during a previous study [5] from asymptomatic roots of white clover, subterranean clover, winter wheat, and winter and spring vetch (Table 1). Fungal colonies for inoculum production were grown on Coons medium [27] at 23 °C under constant black-light blue fluorescent light (F40; range 315 ± 400 nm with the peak at 365 nm). After 20 days of incubation, spores were scraped off from the agar surface with approx. 15 mL of sterile distilled water using a clean microscope slides and enumerated in the suspension with a Fuchs Rosenthal hemocytometer (Paul Marienfeld GmbH & Co. KG, Lauda-Königshofen, Germany). 

Inoculations, greenhouse growing conditions, and disease severity assessments were performed according to the method described previously [2,5]. Briefly, seeds of both plant species were surface sterilized in 70% ethanol for 5 min, rinsed with distilled water and four seeds planted per 500 mL pots which contained approximately 600 g autoclaved sand. Inoculations were performed following sowing with individual *D. pinodella* isolates at 2 × 10^4^ spores g^−1^ substrate. Control pots were left non-inoculated and irrigated with sterile distilled water. Pots were arranged in a completely randomized design with three replicates and kept in the greenhouse at 19 °C day and 16 °C night temperature, and a photoperiod of 16 h light day^−1^ (provided by 400 W high-pressure sodium lamps). Plants were watered daily with tap water and additionally fertilized with complex N:P:K fertilizer Wuxal Super (8:8:6 + microelements; 100 mg of N L^−1^ of substrate). Twenty-one days after inoculations, plants were removed from the pots, the roots separated from above ground parts, washed under running tap water, and assessed for the severity of external and internal root rot symptoms (rated on a 0–8 scale). Roots collected from each treatment were pooled into one plastic bag and stored at −18 °C before further use. 

Culture-based fungal re-isolations and morphological identifications were performed as described previously [5,17] using four pea and five wheat roots randomly selected from each treatment. The same number of roots representing each treatment was used for DNA extractions and qPCR assay validation in infected plant tissue. Plant genomic DNA was extracted from 60 mg lyophilized tissue following the protocol of Sreelakshmi et al. [28]. The quantities and qualities of DNAs were evaluated using a Nanodrop and stored in TE buffer at −20 °C before use. Each qPCR reaction was performed twice.

### 2.6. Preparation of DNA Standards and PCR Efficiency

DNA standards for the 3 isolate/3 species and the 54 isolate/35 species test panels, and the inoculated root samples from the greenhouse were prepared by separate 10 fold dilutions at concentration ranges from 50 ng μL^−1^ to 5 ng μL^−1^ and 100 ng μL^−1^ to 1 ng μL^−1^ using pure culture DNA extracts from *D. pinodella* isolates FOEP 51.11581 or FOEP 51.11670. The quantities of pathogen DNA were determined by extrapolation against the regression line obtained from 10-fold serial dilutions of the pure fungal DNA of the reference *D. pinodella* isolates [12]. Standard curves were generated with CFX Manager Software version 1.0 (Bio-Rad Laboratories, Hercules, CA, USA) by plotting log values of known quantities of targeted DNA versus the corresponding quantification cycle values (Cq). Amplification efficiency (E) of the real-time PCR assay was calculated from a slope of the regression line according to the equation E= {[10^(−1/slope)^] − 1} × 100.

### 2.7. Evaluation of Assay Limit of Detection and Limit of Quantification in Plant Tissue

In order to evaluate whether the quantification of fungal DNA could be performed efficiently in the plant matrix and to determine the assay limit of detection (LOD) and limit of quantification (LOQ) in plant tissue, a dilution series of DNA of *D. pinodella* strain FOEP 51.11670 was made in DNA extract of healthy pea roots ranging from 10.000 pg µL^−1^ to 0.025 pg µL^−1^. The LOD and LOQ were evaluated in four replicates for the concentrations of 3.1 pg µL^−1^ and higher and, in eight replicates for concentrations of 1.6 pg µL^−1^ and lower. LOD was assessed as the minimal concentrations with positive reads for all repetitions, whereas LOQ was the lowest concentration with a standard deviation of replicates smaller than 0.5 Cq values [29] at  ≤35 cycles. This assessment was done twice, as simplex assay (*D. pinodella*, *TEF-1 alpha* alone) and as duplex assay quantifying *D. pinodella TEF-1 alpha* on the FAM channel and the plant 18S assay in the ROX channel in the same reaction with competition of the two simultaneous reactions. The plant DNA was quantified with qPCR primer probes originally developed for apple (Md, malus × domestica) 18S ribosomal DNA [30], but reacting with many other plant species including pea and wheat.

### 2.8. Data Analysis from Greenhouse Experiment

Data from the greenhouse inoculation experiment were analyzed in R [31]. Prior to the analysis, root rot disease severity data were expressed as a disease severity index (DSI) and distinct aggressiveness classes were assigned to each of the isolates as described previously [5]. These data were subjected to ANOVA (package ‘agricolae’; [32]) separately for each inoculated host (i.e., pea and wheat). Data were assessed if they met the assumptions for ANOVA using the Shapiro–Wilk and Levene tests (package ‘car’; [33]) and further verified by verifying if the data contained potentially significant outliers (package ‘outliers’; [34]) and visually inspecting the data normality using the quantile–quantile plots (package ‘ggpur’; [35]). When necessary, raw data were log10 transformed prior to the analysis. If significant isolate effects on fresh weight biomass or DSI were observed, mean values were separated with Tukey HSD test (*p* < 0.05) [32]. A Pearson correlation was performed to determine if there was a relationship between DSI and fresh plant biomass, as well as DSI or fresh plant biomass and DNA quantities of *D. pinodella* in roots averaged over two technical replicates (package ‘stats’; [31]). 

## 3. Results

### 3.1. Primer and Probe Specificity

In the analysis of the forward and reverse primers specificity using a SYBR green assays on a 3 isolates/3 species exclusion panel, all seven primer pairs amplified the target *D. pinodella* but also cross-reacted with the genomic DNA of *D. pinodes* and *D. lethalis*. The melting temperature peak and the Cq values of the product amplified from the target species were similar to that of both non-targets (Table S2).

The primer pair *DpinodellaTef* targeting *TEF-1 alpha* gene selected for the insertion of LNA probe (Table 2) demonstrated high specificity to *D. pinodella* and no cross reactivity with *D. pinodes* and *D. lethalis* when evaluated on a 3 isolates/3 species exclusion panel (Table 3). The assay was specific with annealing/elongation temperatures above 60 °C. When *DpinodellaTef* was tested against the genomic DNAs from 35 non-target organisms (the 54 isolate/35 species panel) cross-reactivity with DNA extracts of five *Didymella* species, namely *D. heteroderae*, *D. aurea*, *D. microchlamydospora*, *D. protuberans* and *D. americana* was observed. However, the non-targets were amplified with at least 10 Cq values higher than *D. pinodella*. The reference strain Cq values ranged from 18 to 19 for a DNA concentration of 100 ng/µL, whereas DNA with the same concentration of non-target species generated signals at Cq 28 for *D. heteroderae* and Cq 34 for *D. americana* (Table 4), hence they were detected with a more than 10^3^ and 10^5^ fold lower sensitivity, respectively. No amplicons were generated with the genomic DNA of strains of the remaining non-target *Didymella* species or other species in the genus *Fusarium* (Table 4).

### 3.2. Sensitivity Analysis, Limit of Detection, and Limit of Quantification of the Assay

**Figure 1:**
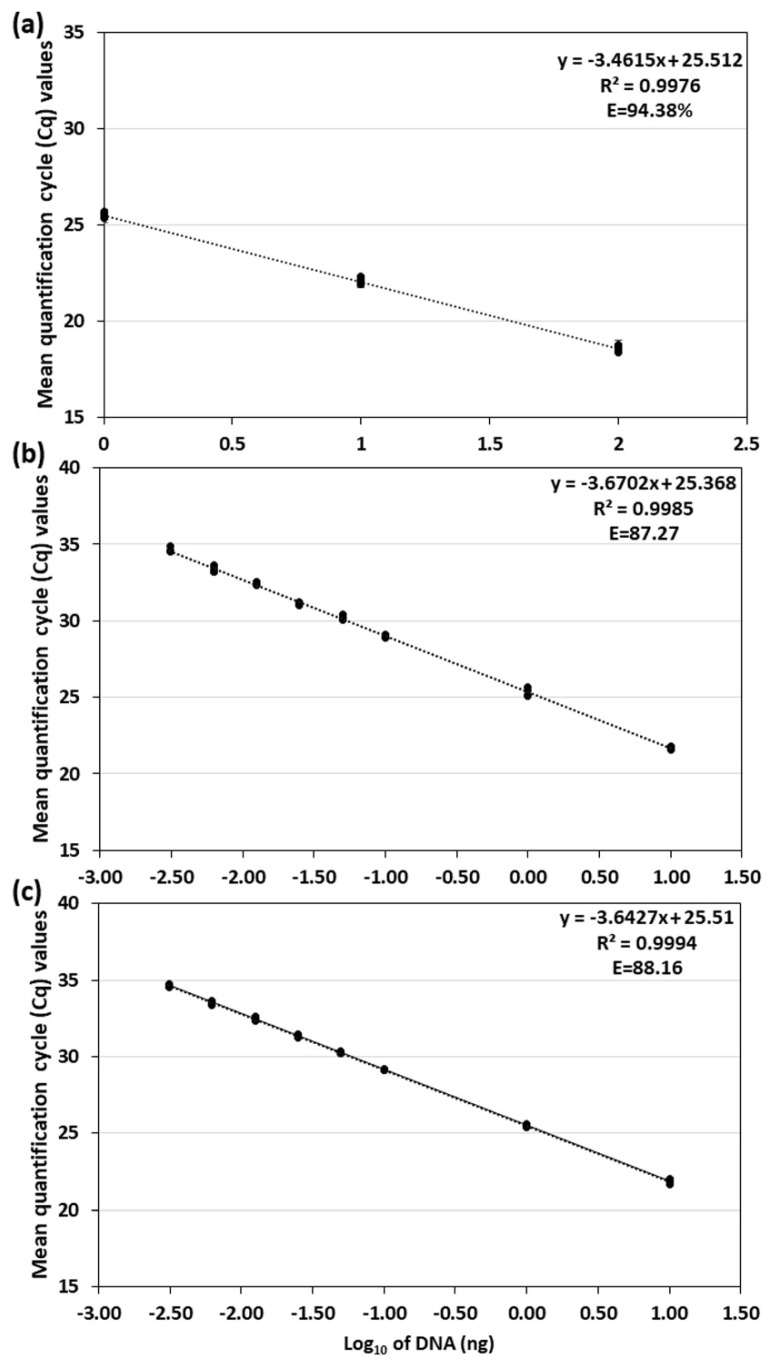
Standard curves for *DpinodellaTef* primer and probe set generated from qPCR assays in this study. Panel (**a**) represents the linear standard curve and the assay performance averaged over five separate qPCR reactions (each performed with two technical replicates) generated in the primer and probe set validation experiments using 10-fold dilution series of *D. pinodella* genomic DNA ranging from 1 to 100 ng (see Table S2 for detailed results); Panels (**b**,**c**) show linear standard curves and the assay performance for the  ≤35 quantification cycles threshold generated for simplex (**b**) and duplex (**c**) qPCR assay used to determine limits of detection (LOD) and quantification (LOQ) of the pathogen DNA in plant tissue. The LOD and LOQ were evaluated for dilution series of *D. pinodella* genomic DNA ranging from 2.5 × 10^−5^ to 10 ng (each in 4 or 8 replicates) made in DNA extract of healthy pea roots (see Table S3 for detailed results). The Cq values are plotted against the DNA concentrations expressed on a logarithmic scale. The error bars were too small to illustrate.

To evaluate the sensitivity of the assay, serial dilutions of *D. pinodella* genomic DNA were analyzed in five separate qPCR reactions. A linear response was observed over 10 fold serial dilutions of pure fungal DNA from 100 ng to 1 ng in all five qPCR reactions (Figure 1a; Table S3). Standard curves calculated from a slope of a regression line had reaction efficiencies (E) which ranged from 92% to 95% and correlation coefficients (R^2^) of 0.99–1.00 (linear regression slope values −3.42 to −3.52). Inter-assay variation determined by reproducibility of standards indicated no inhibition of the target amplification and consistency of the results (Table S3).

The detection limit (LOD) and the limit of quantification (LOQ) of *D. pinodella* DNA in plant tissue in both, simplex and duplex assays, were 3.1 pg of target DNA per reaction (corresponding to 37.4 pg of pathogen DNA/mg dried plant tissue) detected approximately at Cq 35 (mean Cq was 34.63 and standard deviation ± 0.11). Both assays demonstrated similar efficiencies and correlation coefficients. In the simplex assay, the R^2^ was 0.998 and the efficiency of the standard curve 87.3% (slope value −3.67) while these values were slightly different in the duplex qPCR i.e., R^2^ = 0.999 and E = 88.2% (slope value −3.64) (Figure 1b,c; Table S4). 

### 3.3. Greenhouse Experiment—Validation Assay

**Figure 2:**
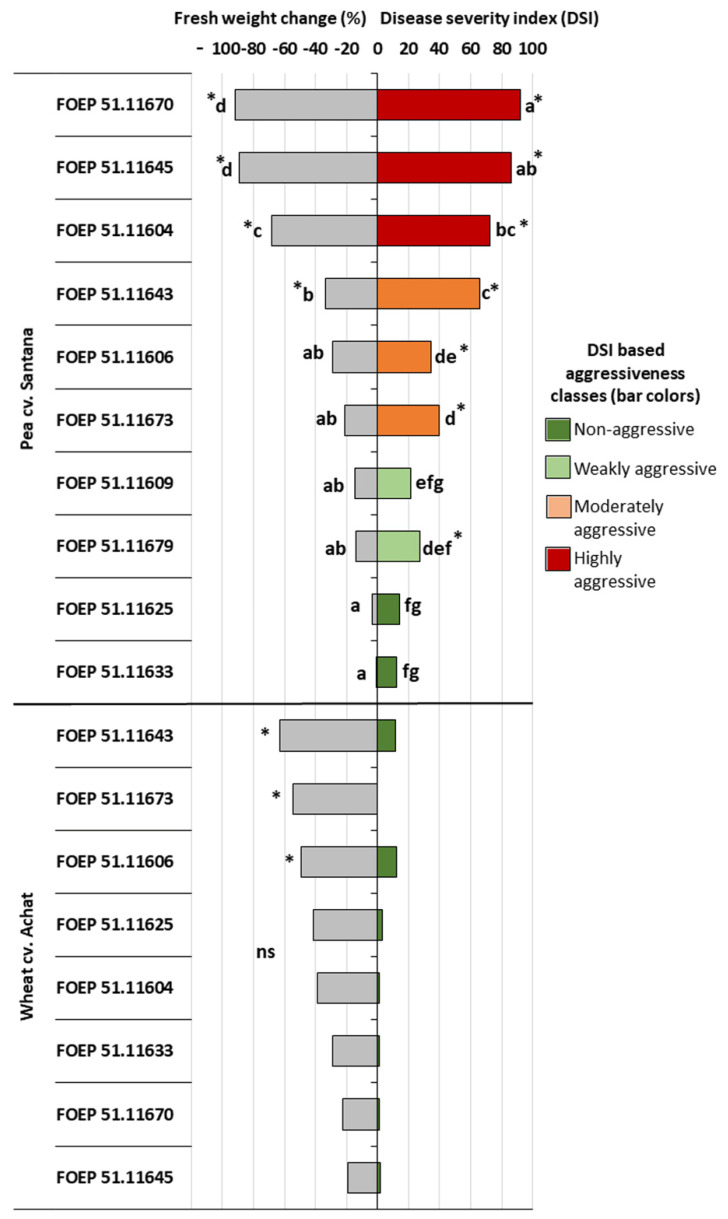
Effects of *D. pinodella* isolates on pea and wheat root rot disease severity (**right**) and plant fresh weight (**left**). The isolate effects on fresh weight are expressed as percentage change relative to the non-inoculated control. Root rot disease severity is expressed as isolate disease severity index (DSI) and corresponds to different bar colors, where DSI = 0–15 non-aggressive; DSI = 16–30 weakly aggressive; DSI = 31–70 moderately aggressive; DSI = 71–100 highly aggressive isolate. Different letters indicate significant differences among isolates (ns = non-significant). Asterisks next to the bars (*) indicate significant difference from the non-inoculated control plants (Tukey multiple comparisons test (*p* < 0.05)). Pea fresh weight data were Log_10_ transformed prior to analysis. Data presented are means of three replicate pots.

**Figure 3:**
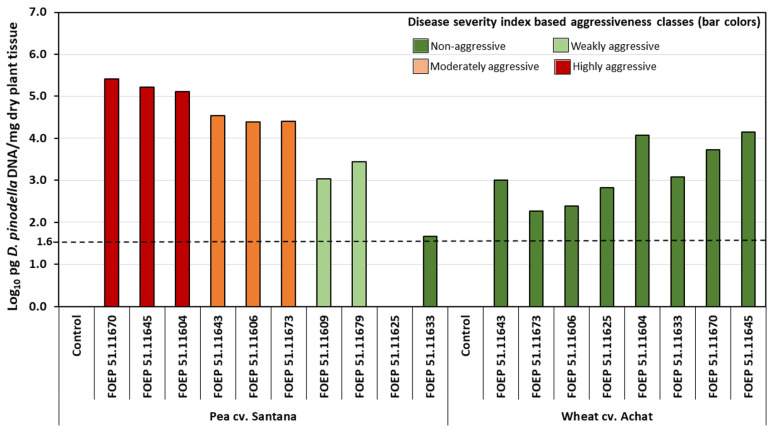
Mean DNA concentrations of *D. pinodella* isolates in greenhouse inoculated pea and wheat roots. The dashed horizontal line shows the assay limit of detection (LOD) which is the minimum amount of the pathogen DNA detectable for the lowest pathogen concentration with positive reads for all repetitions at the ≤35 cycle threshold. Different bar colors correspond to the isolate disease severity index (DSI) classes, where DSI = 0–15 non-aggressive; DSI = 16–30 weakly aggressive; DSI = 31–70 moderately aggressive; DSI = 71–100 highly aggressive. The concentrations are means of two technical replicates expressed on logarithmic scale per milligram (mg) freeze dried root tissue.

Significant variation among individual isolates of *D. pinodella* occurred for both, severity of pea root rot symptoms and pea biomass reduction (Figure 2). Disease severity indices ranged from 12 to 92, and biomass reductions between 1% and 92% compared to the non-inoculated control. Among the 10 isolates tested, two isolates were classified as non-aggressive, two as weakly aggressive, and three isolates each were classified as moderately and highly aggressive, respectively. Four isolates induced significant biomass reduction compared to the non-inoculated control, and these included one weakly aggressive and all moderately and highly aggressive isolates (Figure 2). Root rot severity and pea biomass were highly significantly correlated (Pearson correlation coefficient r = −0.96, *p* < 0.001). The qPCR assay confirmed the presence of *D. pinodella* in all but one of the inoculated treatments (e.g., isolate FOEP 51.11625; Figure 3). The quantities of the pathogen DNA increased linearly with the increase in isolate aggressiveness level (r = 0.84, *p* = 0.001) over a range from 47 pg per mg dried tissue for the non-aggressive isolate FOEP 51.11633 to 2.6 × 10^5^ pg/mg dried tissue for the highly aggressive isolates FOEP 51.11670 (Figure 3). A high and significantly negative correlation between pea fresh weight and *D. pinodella* DNA quantities in pea roots was also observed (r = −0.79, *p* = 0.004). The results from culture dependent methods confirmed the presence of all isolates in pea roots including the isolate FOEP 51.11625 which was below the detection limit in the qPCR assay. However, the qPCR assay also indicated the presence of low quantities of *D. pinodella* isolate FOEP 51.11625 in pea roots but the signal was detected at Cq 36.2.

In contrast to pea, none of the isolates induced symptoms of root rot on wheat (Figure 2). Interestingly, however, despite the absence of root rot symptoms, inoculation with all *D. pinodella* isolates led to reduced wheat biomass, and three isolates (FOEP 51.11606, FOEP 51.11673 and FOEP 51.11643) caused significant biomass reductions compared to the non-inoculated control. Among individual isolates, there was no significant difference in their effect on wheat biomass (Figure 2). All eight *D. pinodella* isolates were detected in inoculated wheat roots with the qPCR assay (Figure 3) and their presence was also confirmed by cultural methods. The quantities of pathogen DNA as determined by qPCR ranged from 1.8 × 10^2^ to 1.4 × 10^4^ pg/mg dried wheat root tissue (Figure 3). There was no correlation between DSI and fresh weight of wheat and/or quantities of pathogen DNA in wheat roots (r_DSI-pathogen DNA_ = 0.03, *p* = 0.93; r_biomass-pathogen DNA_ = −0.29, *p* = 0.45).

## 4. Discussion

This study was prompted by reports which indicated that *D. pinodella* is an important part of the pea root rot complex in Germany, Denmark, and Sweden [2,8,14,36]. Recently, we also found high abundance of this species in symptomatic and asymptomatic pea roots from France and Hungary (Šišić et al., unpublished data) as well as in asymptomatic faba bean, vetch, clover, and wheat roots grown in different regions of Europe [5,14,15]. Given the limitations of culture based methods often coupled with the labor-intensive PCR amplifications, Sanger sequencing and phylogenetic studies, we designed and validated a novel probe-based qPCR assay for detecting and quantification of this pathogen. *Didymella pinodella* can attack both roots and epicotyl (i.e., the foot region) and can cause severe damage and plant death under favorable environmental conditions [9]. In addition to being part of the foot and root rot pathogen complex, *D. pinodella* is also a seed borne pathogen [37] and an important component of the *Ascochyta* blight complex which is a serious disease of peas worldwide [38]. This pathogen can survive as mycelium on infested plant debris and for at least five years as chlamydospores in soil, making rotations in heavily infected areas often of limited success [9]. Davidson et al. [22] previously designed a qPCR assay; however, it could not distinguish *D. pinodella* from *D. pinodes*. Although not evaluated by Davidson et al. [22], their primers and probe set would likely cross-react with *D. lethalis* as the species has an identical ITS sequence with the former two species. In addition to a high level of morphological and genetic similarity, *D. pinodella*, *D. pinodes*, and *D. lethalis* occupy similar ecological niches and can occur together in plant and environmental samples [17,38].

While our assay could provide reliable discrimination of the targeted *D. pinodella* from closely related *D. pinodes* and *D. lethalis*, several other non-targeted *Didymella* spp. gave positive signals. These included *D. heteroderae*, *D. aurea*, *D. microchlamydospora*, *D. protuberans,* and *D. americana*. The risk of cross-contamination, however, is low and unlikely to occur as none of the species were previously associated with pea roots, and only *D. americana* was reported in association with other grain legumes (beans and soybean) and cereals (maize, wheat and millet) [16]. Nevertheless, even if potential co-infections with non-targets occur, it will likely have negligible effects on the results as the amplification levels were up to 10^5^ fold weaker compared with the targeted species. In contrast, no cross-reactivity with any of the relevant *Fusarium* spp. was observed. Furthermore, we show that developing a *D. pinodella*-specific qPCR assay is challenging, and that none of the current housekeeping genes in the *Didymella* genus are suitable for designing *D. pinodella*-specific primer pair due to the very high homology between *D. pinodella*, *D. pinodes* and *D. lethalis* gene sequences. Our results, in contrast, point to a high resolution power of the *TEF-1 alpha* gene region.

The assay was highly consistent across seven separate qPCR reactions and had a quantification/detection limit (LOD and LOQ) of 3.1 pg of target DNA per reaction in plant tissue. It is possible, however, that the LOD and LOQ values are slightly lower than reported here and fall in the range between 1.6 pg µL^−1^ reaction (the detection limit for both simplex and duplex assays at mean Cq 35.76 ± 0.51) and 3.1 pg µL^−1^ per reaction, but we did not pursue these analyses further. This is because the results from greenhouse tests showed that pea root rot symptoms and pea and wheat biomass reductions following pathogen inoculations occurred at *D. pinodella* quantification levels well above 1 ng of pathogen DNA in root samples indicating that the amounts of 3 pg or lower in plant and environmental samples are likely of no significance for plant health. 

The assay enabled detection and quantification of *D. pinodella* in symptomatic and asymptomatic pea and wheat roots. The results from greenhouse experiments further revealed the existence of natural variability in aggressiveness in the population of *D. pinodella* and a strong positive correlation between the quantities of the pathogen DNA in pea roots and the severity of root rot disease symptoms and pea biomass reduction. These results suggest that the aggressiveness of *D. pinodella* to pea may be related to the ability of individual isolates to overcome plant resistance after initial infections. Previous studies have shown, for example, that the aggressiveness of *Fusarium solani* f. sp. *pisi* (syn. *F. pisi*, *F. vanettenii*) towards pea is related to the pathogens ability to synthesize the enzyme pisatin demethylase responsible for degradation of the pea phytoalexin pisatin. All naturally occurring isolates without this ability were essentially non-pathogenic [39]. Moreover, as none of the *D. pinodella* isolates included in this study originated from pea, it appears that various hosts can be asymptomatically colonized by strains of this pathogen which are highly aggressive to pea and thus, these alternative hosts may contribute to the possible spread of this pathogen acting as a reservoir and source of inoculum. Furthermore, although wheat showed no symptoms of root rot following inoculations with any of the *D. pinodella* isolates, all isolates reduced wheat biomass, three causing significant biomass reductions in comparison to the non-inoculated control. Schulz and Boyle [40] previously postulated that endophytic interactions are asymptomatic in their nature and are the result of a balanced antagonism between a microorganism and a host plant. It is thus possible that in the case of the wheat-*D. pinodella* interaction, a higher investment from the plant host is necessary to maintain balanced antagonism at the expense of plant growth. Molecular diagnostic assays, such as the one presented here, are therefore of increasing importance to better understand the relevance of alternative hosts of pathogens as well as the nature of asymptomatic infections and possible implications these may have for control strategies and productivity. The potential of qPCR detection methods to reveal asymptomatic infections and as a tool for epidemiological studies was also demonstrated in other fungus/plant pathosystems [41].

In conclusion, the probe-based qPCR assay described is a reliable procedure for quantification and evaluation of infections caused by *D. pinodella* in different plant hosts. This analytic method could provide further insights into the complex life style of *D. pinodella* and can assist in better understanding the pathogen survival, activity, and spread in the environment. Our results also highlight the need for studies investigating the factors which lead from asymptomatic to symptomatic interactions and indicate that a positive qPCR signal from asymptomatic plants may be relevant for a given plant’s productivity. Further research should also explore the possibilities for multiplexing the assay presented here with the existing assays for the detection and quantification of major pathogenic species associated with pea and other legumes.

## Figures and Tables

**Table 1:** List of fungal strains used in this study. Different species are highlighted in bold.

n ^1^	Species	Isolate ^2^	Host/Substrate	Origin
1	***Boeremia exigua***	FOEP 51.11636	*Vicia villosa*	Sweden
2	***B. exigua***	FOEP 51.11552	*Prunus* sp.	Germany
3	***Didymella americana***	CBS 185.85	*Zea mays*	USA
4	***D. anserina***	CBS 397.65	Plastic	Germany
5	***D. aurea***	CBS 269.93	*Medicago polymorpha*	New Zealand
6	***D. boeremae***	CBS 109942	*Medicago littoralis* seed	Australia
7	***D. exigua***	CBS 183.55	*Rumex arifolius*	France
8	***D. glomerata***	CBS 528.66	*Chrysanthemum* sp.	Netherlands
9	***D. heteroderae***	CBS 109.92	Food	Netherlands
10	***D. lethalis***	FOEP 51.11668	*Vicia villosa*	Italy
11	*D. lethalis*	FOEP 51.11595	*Trifolim subterraneum*	Switzerland
12	*D. lethalis*	FOEP 51.11597	*Trifolim subterraneum*	Switzerland
31	*D. lethalis*	FOEP 51.11588	*Pisum sativum*	Germany
32	*D. lethalis*	FOEP 51.11584	*Pisum sativum*	Germany
33	***D. macrostoma***	FOEP 51.11637	*Vicia villosa*	Switzerland
13	*D. macrostoma*	FOEP 51.11626	*Vicia sativa*	Germany
14	*D. macrostoma*	FOEP 51.11551	*Prunus* sp.	Germany
15	***D. maydis***	CBS 588.69	*Zea mays*	USA
16	***D. microchlamydospora***	CBS 105.95	*Eucalyptus* sp.	UK
17	***D. nigricans***	CBS 444.81	*Actinidia chinensis*	New Zealand
18	***D. pedeiae***	CBS 124517	*Schefflera elegantissima*	Netherlands
19	***D. pinodella***	FOEP 51.11581	*Pisum sativum*	Germany
20	*D. pinodella*	FOEP 51.11606	*Subterranean clover*	Germany
21	*D. pinodella*	FOEP 51.11604	*Subterranean clover*	Germany
22	*D. pinodella*	FOEP 51.11670	*Triticum aestivum*	Germany
23	***D. pinodes***	FOEP 51.11583	*Pisum sativum*	Germany
24	*D. pinodes*	FOEP 51.11590	*Pisum sativum*	Germany
25	*D. pinodes*	FOEP 51.11585	*Pisum sativum*	Germany
26	***D. pomorum***	CBS 539.66	*Polygonum tataricum*	Netherlands
27	***D. protuberans***	CBS 381.96	*Lycium halifolium*	Netherlands
28	***D. subglomerata***	CBS 110.92	*Triticum gramineae*	USA
29	***D. tanaceti***	FOEP 51.11629	*Vicia sativa*	Germany
30	*D. tanaceti*	FOEP 51.11664	*Trifolium repens*	Germany
34	***Didymella*** **sp.**	FOEP 51.11623	*Trifolim subterraneum*	Italy
35	*Didymella* sp.	FOEP 51.11624	*Trifolim subterraneum*	Italy
36	***Fusarium acuminatum***	FOEP 40.11161	*Vicia faba*	Germany
37	***F. avenaceum***	FOEP 11164.1	*Pisum sativum*	Germany
38	***F. crookwellense***	FOEP 40.11152.2	*Vicia faba*	Germany
39	***F. culmorum***	FOEP 40.11152.1	*Pisum sativum*	Germany
40	***F. equiseti***	FOEP 40.11147.1	*Vicia faba*	Germany
41	***F. flocciferum***	FOEP 144.16	*Vicia faba*	Germany
42	***F. graminearum***	FOEP 40.11189.1	*Vicia faba*	Germany
43	***F. oxysporum*** **f. sp. *pisi***	FOEP 40.11162	*Vicia faba*	Germany
44	***F. redolens***	FOEP 40.11140.1	*Pisum sativum*	Germany
45	***F. solani*** **f.sp. *pisi***	FOEP 40.21	*Trifolim subterraneum*	Germany
46	*F. solani* f.sp. *pisi*	FOEP 40.11222	*Pisum sativum*	Germany
47	*F. solani* f.sp. *pisi*	FOEP 40.11169	*Vicia faba*	Germany
48	***F. sporotrichioides***	FOEP 40.11159	*Pisum sativum*	Germany
49	***F. tricinctum***	FOEP 40.11223	*Pisum sativum*	Germany
50	***Juxtiphoma eupyrena (*** **syn. *Phoma eupyrena)***	FOEP 51.11656	*Trifolium repens*	Sweden
51	*J. eupyrena*	FOEP 51.11558	*Vicia faba*	Germany
52	*J. eupyrena*	FOEP 51.11571	*Pisum sativum*	Germany
53	***Paraphaeosphaeria sporulosa***	FOEP 51.11662	*Trifolium repens*	Germany
54	*P. sporulosa*	FOEP 51.11639	*Vicia villosa*	Sweden
55	*D. pinodella* (GH-test)	FOEP 51.11643	*Trifolium repens*	Germany
56	*D. pinodella* (GH-test)	FOEP 51.11604	*Trifolim subterraneum*	Germany
57	*D. pinodella* (GH-test)	FOEP 51.11645	*Trifolium repens*	Germany
58	*D. pinodella* (GH-test)	FOEP 51.11606	*Trifolim subterraneum*	Germany
59	*D. pinodella* (GH-test)	FOEP 51.11670	*Triticum aestivum*	Germany
60	*D. pinodella* (GH-test)	FOEP 51.11673	*Triticum aestivum*	Germany
61	*D. pinodella* (GH-test)	FOEP 51.11625	*Vicia sativa*	Germany
62	*D. pinodella* (GH-test)	FOEP 51.11679	*Triticum aestivum*	Germany
63	*D. pinodella* (GH-test)	FOEP 51.11609	*Trifolim subterraneum*	Germany
64	*D. pinodella* (GH-test)	FOEP 51.11633	*Vicia villosa*	Sweden

^1^ Total number of isolates. Pure fungal DNA test panel included the strains 1–54; qPCR validation assay using a DNA extracts from greenhouse infected plant tissue (GH-test) was performed with the strains 55–64. ^2^ CBS = The Westerdijk Fungal Biodiversity Institute, Utrecht, The Netherlands; FOEP = Culture Collection of the Ecological Plant Protection Department at University of Kassel.

**Table 2:** Nucleotide sequences of *DpinodellaTef* primer and probe set used in the qPCR assay.

Primer/Probe Name	Sequence (5′ to 3′) ^1^	GC%	Amplicon Length (bp)
*DpinodellaTef_forward*	GCACCATGACTTCCTCCA	56	78
*DpinodellaTef_reverse*	CCTGTAATGATTGTTAGCTTTATGA	32	
*DpinodellaTef_probe*	FAM-TGGCAC**[TAT]**TGTCGCATTCTCACT–BHQ1	46	

^1^ The position of the locked nucleic acid (LNA) modified bases in the probe sequence are shown in square brackets and highlighted in bold.

**Table 3:** Quantification cycle (Cq) values of *DpinodellaTef* primer and probe set evaluated in the specificity assay on a 3 isolates/3 species exclusion panel. The optimum primer annealing temperatures and two different fungal DNA concentrations were also tested.

Annealing Temp. (°C)	DNA Concentration (ng/µL)	*D. pinodella* Cq ^1^	*D. pinodes* Cq	*D. lethalis* Cq	H_2_O Cq
62.0	5	28.19	-	-	n/a
62.0	50	24.42	-	-	n/a
60.7	5	28.15	-	-	n/a
60.7	50	24.21	-	-	n/a
59.1	5	28.3	34.27	-	n/a
59.1	50	24.7	30.31	-	n/a
57.2	5	29.16	30.84	-	n/a
57.2	50	25.34	27.44	-	n/a
55.6	5	29.4	30.17	-	n/a
55.6	50	26.19	26.05	-	n/a
54.0	5	30.08	30.11	34.83	n/a
54.0	50	26.29	27.02	31.19	n/a
61.6	H_2_O	n/a	n/a	n/a	37.79
54.5	H_2_O	n/a	n/a	n/a	-

^1^ n/a—not tested. “-” = no signal was detected.

**Table 4:** Quantification cycle (Cq) values of *DpinodellaTef* primer and probe set tested against pure fungal DNA extracts in a 54 isolate/35 species validation panel.

Species	Isolate	Cq ^1^
*Didymella pinodella*	FOEP 51.11670	18.58
*D. pinodella*	FOEP 51.11606	18.61
*D. pinodella*	FOEP 51.11604	19.27
*D. heteroderae*	CBS 109.92	28.25
*D. microchlamydospora*	CBS 105.95	30.23
*D. protuberans*	CBS 381.96	31.02
*D. aurea*	CBS 269.93	31.62
*D. americana*	CBS 185.85	34.34
*Boeremia exigua*	FOEP 51.11636	-
*B. exigua*	FOEP 51.11552	-
*D. anserina*	CBS 397.65	-
*D. boeremae*	CBS 109942	-
*D. exigua*	CBS 183.55	-
*D. glomerata*	CBS 528.66	-
*D. lethalis*	FOEP 51.11584	-
*D. lethalis*	FOEP 51.11588	-
*D. lethalis*	FOEP 51.11595	-
*D. lethalis*	FOEP 51.11597	-
*D. lethalis*	FOEP 51.11668	-
*D. macrostoma*	FOEP 51.11626	-
*D. macrostoma*	FOEP 51.11551	-
*D. macrostoma*	FOEP 51.11637	-
*D. maydis*	CBS 588.69	-
*D. nigricans*	CBS 444.81	-
*D. pedeiae*	CBS 124517	-
*D. pinodes*	FOEP 51.11583	-
*D. pinodes*	FOEP 51.11585	-
*D. pinodes*	FOEP 51.11590	-
*D. pomorum*	CBS 539.66	-
*D. subglomerata*	CBS 110.92	-
*D. tanaceti*	FOEP 51.11664	-
*D. tanaceti*	FOEP 51.11629	-
*Didymella* sp.	FOEP 51.11624	-
*Didymella* sp.	FOEP 51.11623	-
*Fusarium acuminatum*	FOEP 40.11161	-
*F. avenaceum*	FOEP 11164.1	-
*F. crookwellense*	FOEP 40.11152.2	-
*F. culmorum*	FOEP 40.11152.1	-
*F. equiseti*	FOEP 40.11147.1	-
*F. flocciferum*	FOEP 144.16	-
*F. graminearum*	FOEP 40.11189.1	-
*F. oxysporum* f. sp. *pisi*	FOEP 40.11162	-
*F. redolens*	FOEP 40.11140.1	-
*F. solani* f. sp. *pisi*	FOEP 40.21	-
*F. solani* f. sp. *pisi*	FOEP 40.11169	-
*F. solani* f. sp. *pisi*	FOEP 40.11222	-
*F. sporotrichioides*	FOEP 40.11159	-
*F. tricinctum*	FOEP 40.11223	-
*Juxtiphoma eupyrena* (syn. *Phoma eupyrena*)	FOEP 51.11558	-
*J. eupyrena*	FOEP 51.11571	-
*J. eupyrena*	FOEP 51.11656	-
*Paraphaeosphaeria sporulosa*	FOEP 51.11639	-
*P. sporulosa*	FOEP 51.11662	-

^1^ Cq values are means from two separate qPCR reactions and two technical replicates per reaction. Cq value for *D. pinodella* FOEP 51.11670 (CPC 28850) is the mean of ten technical replicates of five separate reactions. “-” = Cq below limit of detection.

## Data Availability

All relevant data generated or analyzed during this study are included in this article.

## Supplementary Materials

The following supporting information can be downloaded at: https://www.mdpi.com/article/10.3390/jof8010041/s1, Figure S1: *DpinodellaTef qPCR* primer and probe specific for *Didymella pinodella.*; Table S1: Candidate primer pairs designed in silico and tested for specificity in the initial SYBR green assays on a 3 isolates/3 species exclusion panel. Highlighted in red are single nucleotide polymorphs of the target primer and probe sequence and the two closely related *Didymella* spp.; Table S2. Quantification cycle (Cq) values and the melt curve data (Tmelt) for *DpinodellaTef* primer pair specificity tests in a SYBR green assay on a 3 isolates/3 species exclusion panel. In subsequent analysis this primer pair was selected for the insertion of LNA probe. The primer pair was tested using two different fungal DNA concentrations (5 and 50 ng/microL) and under temperature gradient from 55 to 63 °C. The remaining six primer pairs (Table S1) were evaluated under the same conditions and yielded similar results e.g., amplified the target *D. pinodella* but also cross-reacted with the genomic DNA of *D. pinodes* and *D. lethalis*.; Table S3: Inter-assay comparison of performance of *DpinodellaTef* primer and probe set across five separate qPCR assays each containing two technical replicates. Cq = quantification cycle values.; Table S4: Evaluation of limit of detection (LOD) and limit of quantification (LOQ) in plant samples and performance of *DpinodellaTef* primer and probe set in simplex (*DpinodellaTef* alone) and duplex (*DpinodellaTef* on the FAM channel and the plant 18S assay in the ROX channel) qPCR assay. The reactions were performed in four replicates for the concentrations of 3.1 pg µL^−1^ and higher and eight replicates for concentrations of 1.6 pg µL^−1^ and lower. Cq = quantification cycle values; CV = coefficient of variation.

## References

[1] Arias M.D., Munkvold G.P., Ellis M.L., Leandro L.F.S. (2013). Distribution and Frequency of *Fusarium* species Associated with Soybean Roots in Iowa. Plant Dis..

[2] Baćanović-Šišić J., Šišić A., Schmidt J.H., Finckh M.R. (2018). Identification and Characterization of Pathogens Associated with Root Rot of Winter Peas Grown under Organic Management in Germany. Eur. J. Plant Pathol..

[3] Chatterton S., Harding M.W., Bowness R., Mclaren D.L., Banniza S., Gossen B.D. (2019). Importance and Causal Agents of Root Rot on Field Pea and Lentil on the Canadian Prairies, 2014–2017. Can. J. Plant Pathol..

[4] Chittem K., Mathew F.M., Gregoire M., Lamppa R.S., Chang Y.W., Markell S.G., Bradley C.A., Barasubiye T., Goswami R.S. (2015). Identification and Characterization of *Fusarium* spp. Associated with Root Rots of Field Pea in North Dakota. Eur. J. Plant Pathol..

[5] Šišić A., Baćanović-Šišić J., Karlovsky P., Wittwer R., Walder F., Campiglia E., Radicetti E., Friberg H., Baresel J.P., Finckh M.R. (2018). Roots of Symptom-Free Leguminous Cover Crop and Living Mulch Species Harbor Diverse *Fusarium* Communities That Show Highly Variable Aggressiveness on Pea (*Pisum sativum*). PLoS ONE.

[6] Gaulin E., Bottin A., Jacquet C., Dumas B., Lamour K., Kamoun S. (2009). Aphanomyces euteiches and Legumes. Oomycete Genetics and Genomics. Diversity, Interactions and Research Tools.

[7] Hwang S.F., Chang K.F. (1989). Incidence and Severity of Root Rot Disease Complex of Field Pea in Northeastern Alberta in 1988. Can. Plant Dis. Surv..

[8] Persson L., Bødker L., Larsson-Wikström M. (1997). Prevalence and Pathogenicity of Foot and Root Rot Pathogens of Pea in Southern Scandinavia. Plant Dis..

[9] Tran H.S., You M.P., Khan T.N., Barbetti M.J. (2016). Pea Black Spot Disease Complex on Field Pea: Dissecting the Roles of the Different Pathogens in Causing Epicotyl and Root Disease. Eur. J. Plant Pathol..

[10] Xue A.G. (2003). Biological Control of Pathogens Causing Root Rot Complex in Field Pea Using *Clonostachys rosea* Strain ACM941. Phytopathology.

[11] Schena L., Nigro F., Ippolito A., Gallitelli D. (2004). Real-Time Quantitative PCR: A New Technology to Detect and Study Phytopathogenic and Antagonistic Fungi. Eur. J. Plant Pathol..

[12] Zitnick-Anderson K., Simons K., Pasche J.S. (2018). Detection and QPCR Quantification of Seven *Fusarium* species Associated with the Root Rot Complex in Field Pea. Can. J. Plant Pathol..

[13] Armstrong-Cho C., Tetreault M., Banniza S., Bhadauria V., Morrall R.A.A. (2014). National Coordinator/Coordinateur National. Can. Plant Dis. Surv..

[14] Šišić A., Baćanović-Šišić J., Schmidt H., Finckh M.R., Brka M., Omanović-Mikličanin E., Karić L., Falan V., Toroman A. (2020). Root Pathogens Occurring on Pea (*Pisum sativum*) and Faba Bean (*Vicia faba*) in Germany. 30th Scientific-Experts Conference of Agriculture and Food Industry: Answers for Forthcoming Challenges in Modern Agriculture.

[15] Šišić A., Baćanović-Šišić J., Finckh M.R. Molecular Characterization and Aggressiveness of *Didymella pinodella* Isolates Associated with Root Rot of Field Pea (*Pisum sativum*). Proceedings of the 61 Deutsche Pflanzenschutztagung “Herausforderung Pflanzenschutz—Wege in die Zukunft”, University Hohenheim.

[16] Farr D.F., Rossman A.Y. Fungal Databases, Systematic Mycology and Microbiology Laboratory, ARS, USDA. http://nt.ars-grin.gov/fungaldatabases/.

[17] Šišić A., Baćanović-Šišić J., Schmidt H., Finckh M.R. (2018). First Report of *Didymella lethalis* Associated with Roots of Pea, Subterranean Clover and Winter Vetch in Germany, Switzerland and Italy. Plant Dis..

[18] Chen Q., Hou L.W., Duan W.J., Crous P.W., Cai L. (2017). *Didymellaceae* Revisited. Stud. Mycol..

[19] Chen Q., Jiang J.R., Zhang G.Z., Cai L., Crous P.W. (2015). Resolving the *Phoma* Enigma. Stud. Mycol..

[20] Bowen J.K., Lewis B.G., Matthews P. (1997). Discovery of the Teleomorph of *Phoma medicaginis var. pinodella* in Culture. Mycol. Res..

[21] Aveskamp M.M., Verkley G.J.M., de Gruyter J., Murace M.A., Perello A., Woudenberg J.H.C., Groenewald J.Z., Crous P.W. (2009). DNA Phylogeny Reveals Polyphyly of *Phoma* Section *Peyronellaea* and Multiple Taxonomic Novelties. Mycologia.

[22] Davidson J.A., Krysinska-Kaczmarek M., Wilmshurst C.J., McKay A., Herdina, Scott E.S. (2011). Distribution and Survival of *Ascochyta* Blight Pathogens in Field-Pea-Cropping Soils of Australia. Plant Dis..

[23] Rigorth K.S., Finckh M., Šišić A. (2021). First Report of *Fusarium venenatum* Causing Foot and Root Rot of Wheat (*Triticum aestivum*) in Germany. Plant Dis..

[24] Šišić A., Baćanović-Šišić J., Al-Hatmi A.M.S., Karlovsky P., Ahmed S.A., Maier W., de Hoog G.S., Finckh M.R. (2018). The ‘Forma specialis’ Issue in *Fusarium*: A Case Study in *Fusarium Solani* f. sp. Pisi. Sci. Rep..

[25] Katoh K., Standley D.M. (2013). MAFFT Multiple Sequence Alignment Software Version 7: Improvements in Performance and Usability. Mol. Biol. Evol..

[26] Letertre C., Perelle S., Dilasser F., Arar K., Fach P. (2003). Evaluation of the Performance of LNA and MGB Probes in 5′-Nuclease PCR Assays. Mol. Cell. Probes.

[27] Coons G.H. (1916). Factors Involved in the Growth and the Pycnidium Formation of *Plenodomus fuscomaculans*. J. Agric. Res..

[28] Sreelakshmi Y., Gupta S., Bodanapu R., Chauhan V.S., Hanjabam M., Thomas S., Mohan V., Sharma S., Srinivasan R., Sharma R. (2010). NEATTILL: A Simplified Procedure for Nucleic Acid Extraction from Arrayed Tissue for Tilling and Other High-Throughput Reverse Genetic Applications. Plant Methods.

[29] Forootan A., Sjöback R., Björkman J., Sjögreen B., Linz L., Kubista M. (2017). Methods to Determine Limit of Detection and Limit of Quantification in Quantitative Real-Time PCR (QPCR). Biomol. Detect. Quantif..

[30] Oberhänsli T., Vorley T., Tamm L., Schärer H.J. (2014). Development of a Quantitative PCR for Improved Detection of Marssonina coronaria in Field Samples.

[31] R Core Team (2013). R: A Language and Environment for Statistical Computing.

[32] Mendiburu D.F. (2014). Agricolae: Statistical Procedures for Agricultural Research.

[33] Fox J., Weisberg S. (2019). An R Companion to Applied Regression.

[34] Komsta L. (2011). Package ‘Outliers’. A Collection of Some Tests Commonly Used for Identifying Outliers.

[35] Kassambara A. (2020). Package ‘Ggpubr’: “ggplot2” Based Publication Ready Plots. https://rpkgs.datanovia.com/ggpubr/.

[36] Pflughöft O., Merker C., von Tiedemann A., Schäfer B.C. (2012). Zur Verbreitung und Bedeutung von Pilzkrankheiten in Körnerfuttererbsen (*Pisum*
*sativum* L.) in Deutschland. Gesunde Pflanz..

[37] Saeed M.F., Baćanović J., Bruns C., Schmidt H., Finckh M.R. (2017). Seed Health of Organic Peas and Faba Beans and Its Effects on the Health of the Harvested Grains. J. Plant Dis. Prot..

[38] Khan T.N., Timmerman-Vaughan G.M., Rubiales D., Warkentin T.D., Siddique K.H.M., Erskine W., Barbetti M.J. (2013). *Didymella pinodes* and Its Management in Field Pea: Challenges and Opportunities. Field Crop. Res..

[39] Hadwiger L.A. (2008). Pea-*Fusarium*
*solani* Interactions Contributions of a System toward Understanding Disease Resistance. Phytopathology.

[40] Schulz B., Boyle C. (2005). The Endophytic Continuum. Mycol. Res..

[41] Schena L., Abdelfattah A., Mosca S., Li Destri Nicosia M.G., Agosteo G.E., Cacciola S.O. (2017). Quantitative detection of *Colletotrichum godetiae* and *C. acutatum sensu stricto* in the phyllosphere and carposphere of olive during four phenological phases. Eur. J. Plant Pathol..

