# Supplementary figures and images for: A Novel Real Time PCR Method for the Detection and Quantification of Didymella pinodella in Symptomatic and Asymptomatic Plant Hosts
Source: J Fungi (Basel). 2021 Dec 31;8(1):41. doi: 10.3390/jof8010041 (PMC8780382; doi:10.3390/jof8010041)

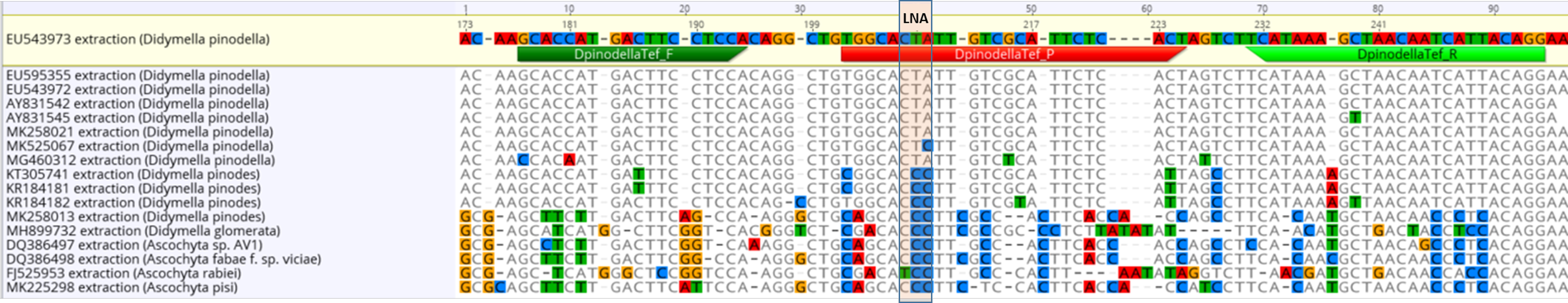

Supplement: Supplementary file 1 [file jof-08-00041-s001.zip › Figure S1_Sisic.tiff]
